# Impact of cognitive impairment on driving behaviour and route choices of older drivers: a real-world driving study

**DOI:** 10.1038/s41598-024-63663-y

**Published:** 2024-06-19

**Authors:** Reihaneh Derafshi, Ganesh M. Babulal, Sayeh Bayat

**Affiliations:** 1 Department of Biomedical Engineering, University of Calgary, Calgary, Canada; 2 Department of Neurology, Washington University School of Medicine, St. Louis, USA; 3 Department of Clinical Research and Leadership, The George Washington University School of Medicine and Health Sciences, Washington, D.C., USA; 4 Department of Psychology, Faculty of Humanities, University of Johannesburg, Johannesburg, South Africa; 5 Department of Geomatics Engineering, University of Calgary, Calgary, Canada; 6 Hotchkiss Brain Institute, University of Calgary, Calgary, Canada; 7 Institute of Public Health, Washington University, St. Louis, USA

**Keywords:** Driving behaviours, Rout choices, Older adults, Dementia, Risk factors, Alzheimer's disease, Alzheimer's disease, Quality of life

## Abstract

Maintaining driving independence is important for older adults. However, cognitive decline, a common issue in older populations, can impair older adults’ driving abilities and overall safety on the roads. This study explores how cognitive impairment influences driving patterns and driving choices among older adults. We analyzed real-world driving patterns of 246 older adults using GPS dataloggers. Our sample included 230 cognitively normal older adults (CN; Clinical Dementia Rating$^R$ [CDR] = 0) and 16 older adults with incident cognitive impairment (ICI; CDR = 0.5). The CN group had an average age of 68.2 years, with 46% females and an average of 16.5 years of education, while the ICI group’s average age was 69.2 years, with 36% females and an average of 16.0 years of education. We employed spatial clustering and hashing algorithms to evaluate driving behaviours. Significant differences emerged: The ICI group used fewer distinct routes to their most common destination. These differences can be leveraged to develop driving as a digital biomarker for the early detection and continuous monitoring of cognitive impairment.

## Introduction

Driving is essential to maintaining independence and social engagement, particularly for older adults^1^. It represents not just a means of transportation but a significant component of personal autonomy and social interaction^2^. However, the aging process brings an increased risk for dementia. Alzheimer’s Disease and Related Dementias (ADRD) are a spectrum of neurodegenerative conditions characterized by progressive cognitive decline and memory loss^3^. ADRD affect various cognitive functions, including memory, attention, language, and problem-solving abilities. The onset of dementia is typically gradual, and its progression varies among individuals^4^. More specifically, dementia can lead to a decline in memory and other cognitive skills, impairing a person’s ability to perform daily activities. This decline can impact driving abilities and eventually lead to the cessation of driving^5^. Such a change can have significant physical, social, and mental health consequences for older adults, leading to social isolation, depression, faster progression of cognitive impairment, and even transition to institutional care^6–9^.

Emerging evidence suggests that everyday driving may serve as a behavioural marker for detecting cognitive decline, with subtle changes manifesting early in the disease progression^10,11^. Previous studies on naturalistic driving and dementia have concentrated on quantifying driving space, such as the radius of gyration, and driving performance metrics, including speed and acceleration, often aggregating these measures over time^12^. These existing studies do not provide a nuanced understanding of the day-to-day driving choices and challenges faced by drivers with cognitive impairments, and overlook the significance of route choices-factors crucial to understanding the full impact of cognitive decline on driving.

Common trips, representing routine and often repeated journeys, are particularly revealing of an individual’s driving habits and are better representatives of their overall driving behaviour^13^. Moreover, the choice of routes, a factor previously neglected in many studies, holds critical importance in the contex of dementia^14,15^. It reflects an individual’s navigational decision-making and their ability to engage with and adapt to their environment, an aspect directly influenced by decline in cognitive abilities.

The importance of this research lies in its potential to inform public health strategies and road safety measures, particularly for an aging population, by emphasizing the complexities of driving needs in older adults with cognitive impairment. By gaining a deeper understanding of how cognitive decline affects driving, interventions and driving aids, such as personalized navigation tools, can be effectively designed to assist older drivers, thereby enhancing their safety and mobility while contributing to overall road safety^16^.

This study aims to bridge the existing gap by employing a novel approach that uses GPS dataloggers to passively monitor the real-world driving patterns of older adults, both cognitively normal and those with impairments. By focusing on navigational decision-making, this study seeks to understand the complex relationship between the frequency of driving on specific routes, the occurrence of driving errors, and the presence of cognitive impairment. Additionally, this study examines gender differences to gain insights into how cognitive decline affects driving behaviours and route choices among older adults.

## Methods

### Participants

The study comprised 246 participants, all of whom were 65 years of age or older, required to drive at least once a week at baseline, have a valid driver’s license, drive a non-adapted vehicle, and agree to annual follow-up. These participants were enrolled in longitudinal studies of aging, driving, and Alzheimer’s disease at Washington University School of Medicine, St. Louis, Missouri, United States^17,18^. The participant group included 230 cognitively normal older adults (CN), characterized by a Clinical Dementia Rating$^R$ (CDR)^19^ of 0, indicating no cognitive impairment. The remaining 16 participants were older adults with incident cognitive impairment (ICI), as evidenced by a CDR score of 0.5. The participation duration for all individuals spanned two years. This study was approved by the WUSTL Institutional Review Board (IRB # 202010214 and 201706043), and each participant provided signed informed consent. All methods were carried out in accordance with relevant guidelines and regulations.

In this study, a commercial GPS data logger, specifically the G2 Tracking Device$^{\tiny \textrm{TM}}$ by Azuga Inc., San Jose, CA, United States, was installed in the personal vehicles of all participants to gather real-time driving data over a 2-year duration. The device recorded a variety of parameters at 30-s intervals, including geographical coordinates (longitude and latitude), date and time stamps, speed, distance covered, and acceleration metrics. The data logger was also equipped with an accelerometer, which captured minor or major impacts, such as hard braking and sudden acceleration (Table 1).

**Table 1:** Demographic characteristics of study participants.

Characteristic	CN (CDR = 0)	ICI (CDR = 0.5)
Number of participants (n)	230	16
Average age (years) (n + SD)	$68.2 \pm 5.7$	$69.2 \pm 6.8$
Female (%)	46	36
Wearing glasses or contacts while driving (%)	71	55
Using hearing aids (%)	34	16
Using their hearing aids while driving (%)	88	56

### Data preprocessing

During the preprocessing phase, we implemented a procedure to clean the dataset. Our preprocessing approach involved three crucial steps. A primary focus was on handling instances of short trips with low movement that lack navigational and route choice information useful to this study and, if not removed, could be misidentified as ’common’ trips. In our analysis, we categorized ’cycle trips’ as those where the vehicle starts and ends at the same location. While start and end points typically provide insights into navigational choices and paths taken, this is not the case with cycle trips. In these instances, such starting and ending locations offer no predictive value regarding the route. As a result, cycle trips can show substantial route variations, even when they have similar endpoints. Therefore, we analyzed these trips separately to account for their unique characteristics. Lastly, we excluded any incomplete trips that did not follow the predefined format of starting and ending from the dataset. These were trips interrupted due to technical issues, such as GPS signal loss. Eliminating these incomplete records helped maintain the integrity of our driving data, ensuring inclusion of only fully recorded trips in subsequent analyses.

### Trip risk score computation

For each trip, we computed a risk score to evaluate driving behaviour. This score integrated incidents of hard braking and acceleration, detected by the datalogger’s accelerometer, as well as speeding and under-speeding, identified through speed and GPS data. We chose to measure these incidents in 2-min duration intervals because this timeframe balances the instantaneous nature of incidents like hard braking and acceleration with the more prolonged nature of speeding and under-speeding errors. Aligned with our 30-s data collection rate, this method allowed for a detailed analysis, providing up to four data points per interval for accuracy. The total occurrences were then normalized by the number of intervals.

In our study, we conducted a sensitivity analysis to validate the threshold for classifying trips as ’risky’. This involved adjusting the original risk score threshold by predetermined percentages both above and below its initial value, creating a series of different thresholds. For each threshold, we recalculated the risk scores for all trips, observing the resulting changes in trip classifications. We systematically analyzed the number and proportion of trips classified as risky under each threshold variant, using statistical methods to assess the stability of these classifications. Minimal changes in classification suggested a reliable threshold, whereas significant variations indicated the need for further refinement of the threshold value.This analysis provided a reliable approach for setting threshold, ensuring that we accurately identify risky trips without falsely categorizing safe trips as risky.

### Algorithm for trip clustering

To analyze individual driving patterns, we developed a custom clustering algorithm tailored for GPS driving data. This hierarchical, trajectory-based method focuses on clustering participant trips based on path similarity, utilizing hashed values and subset matching. The algorithm’s execution on a per-participant basis enabled a thorough examination of various driving characteristics, revealing details in the frequency of routinely taken trips, the diversity of routes to common destinations, the overall count of unique destinations visited, and the count of new destinations. Additionally, it provided insights into changes in driving behaviours in relation to the repetitiveness of routes.

#### Characteristics and steps of the algorithm

We employed hashing and a two-step clustering process to analyze driving routes. For hashing, each coordinate was mapped to a specific area on the map, defined by rounding coordinates to two decimal places, resulting in approximate dimensions of 1.11 km by 0.90 km based on the location of St. Louis. The clustering process was divided into two steps to enhance efficiency and reduce algorithm complexity, subsequently leading to a reduction in computational demand.

First, trips were grouped based on their starting points and destinations, without considering the varied paths taken between these points. These clusters were subsequently sorted by their size.

For the second step, we focused on path similarity within each of the initially formed clusters. This approach allowed us to identify the number of distinct paths for each start-destination pair. We prioritized larger clusters, representing the most common trips, to significantly reduce the computational load. Smaller clusters did not proceed to this second stage of clustering.

For the advanced clustering phase, which constitutes the second step of our clustering process, an improved hashing technique was employed. A unique hashed array was created for each trip. Coordinates were hashed as described, and if the new hash differed from the last for that trip, it was added to the trip’s hashed array, avoiding consecutive duplicates. Hierarchical clustering began with these hashed arrays. A trip was added to a cluster if its hashed array was a subset of the cluster’s representative array or vice versa. If the former occurs, the trip’s hashed array replaces the cluster’s representative array. In other words, the cluster’s representative array is a subset of all its member trips’ hashed arrays.

### Computed mobility metrics

Subsequent to the formation of clusters through our algorithm, we conducted further analyses and computed the following metrics for each participant. Detailed information for each metric, including Total Trips, Destinations, Short Trips, Cycle Trips, New Destinations, Most Common Destination, Most Common Route, Distinct Paths, and Risky Trips, can be found in Table 2.

**Table 2:** Detailed description of the mobility metrics computed after data processing and clustering.

Metric	Description
Total trips	The total number of trips taken by each participant, counted after the preprocessing steps were completed.
Cycle trips	The number of trips that have the same start and end points.
New destinations	This metric counts the pairs of (start-point, destination) that were visited only once during the participation period.
Most common destination	This is the pair of (start-point, destination) that was visited most frequently by each participant.
Most common route	The most frequently used route to the most common destination, Identified in the second step of clustering.
Distinct paths	The number of different routes taken to the most common destination, calculated as the count of clusters in the second step of clustering.
Risky new destination trips	The proportion of new destination trips classified as risky.
Risky most common destination trips	The proportion of most common destination trips classified as risky.
Risky most common route trips	The proportion of most common route trips classified as risky.

Each metric underwent normalization tailored to its specific characteristics. For instance, the number of short trips, cycle trips, new destinations, and the frequency of the most common destination were normalized by dividing by the total number of trips. This normalization method effectively eliminates the influence of overall driving frequency on these events.

Furthermore, the frequency of trips driven by taking the most common route was normalized by dividing by the number of trips to the most common destination. This normalization strategy illustrates the consistency in utilizing this route to reach the destination.

Finally, the number of distinct paths used to the most common destination was normalized by dividing by the average frequency of this trip over a month. This normalization technique captures the willingness to explore various routes for reaching the most common destination while mitigating the impact of repetition of this trip.

### Statistical methods

To determine significant differences between the two participant groups across each computed metric, we initially conducted a Shapiro-Wilk normality test to assess the distribution of our metrics. Given that non of them did not adhere to a normal distribution, we used the nonparametric Mann–Whitney U-test^20^.

The Mann–Whitney U-test can compare distributions of non-normally distributed data between groups, enabling us to identify differences in medians accurately.

Given the large number of comparisons involved in conducting statistical tests of over 8 measures, we used the Bonferroni adjustment to reduce the possible inflation of the Type I error rate. Accordingly, the significance level was adjusted to $\alpha = \frac{0.05}{8} \approx 0.0062$, ensuring the robustness of our statistical analysis and our hypothesis testing.

## Results

**Figure 1:**
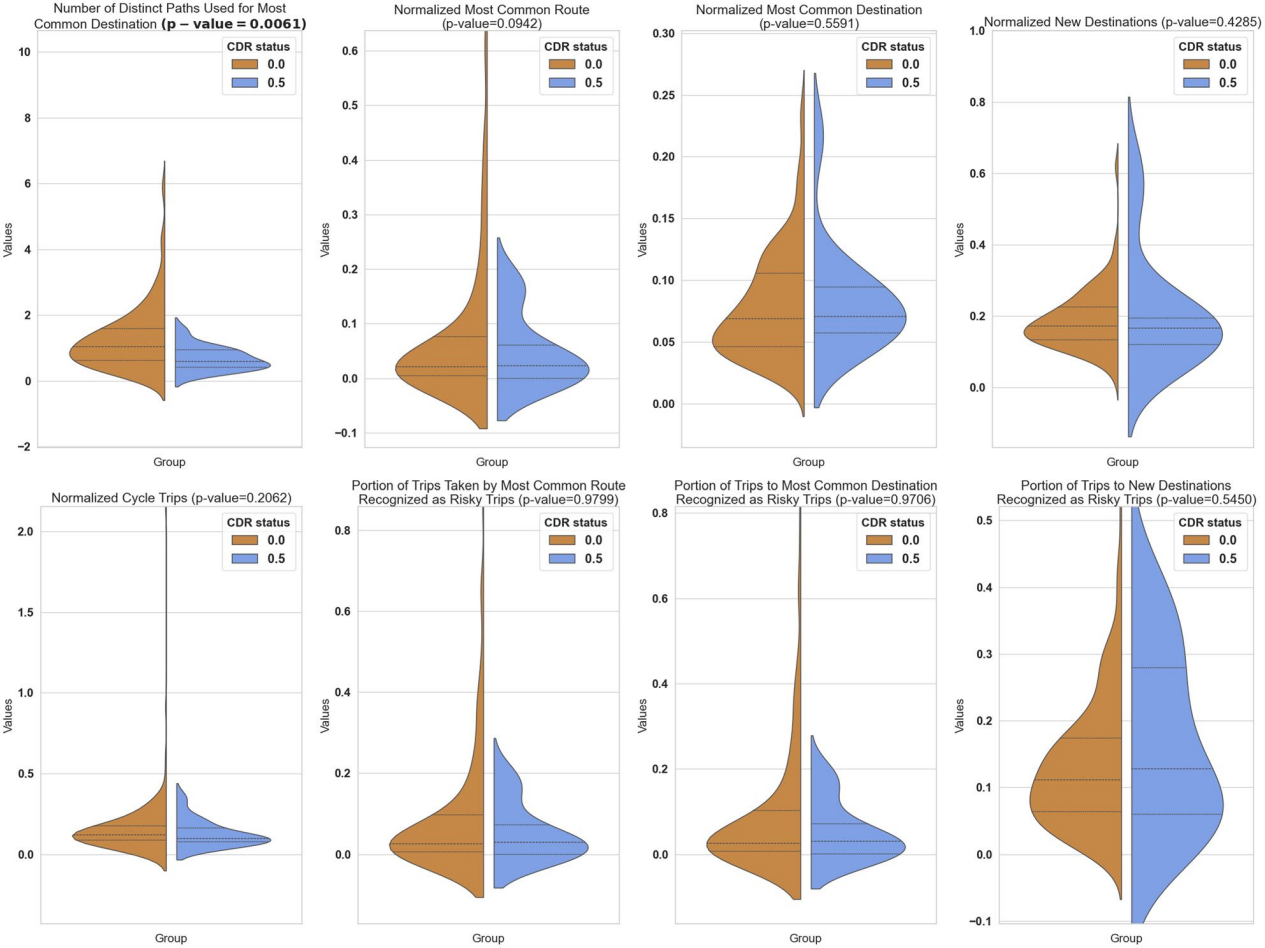
Violin plots comparing the distribution and medians of driving metrics between the CN group with a CDR score of 0.0 and the ICI group with a CDR score of 0.5 are presented. The first figure represents the normalized variety of routes to the most common destination, revealing a significant difference between the two groups with a p-value of 0.0061. The subsequent two figures depict normalized most common routes and normalized most common destinations, both of which are indicative of navigational-based driving behaviours.The following two figures illustrate trips with specific properties, such as normalized new destinations and cycle trips. The subsequent three figures represent the proportion of risky trips for three clusters: most common route, most common destination, and new destinations. No significant differences were observed in the above measures in this study^21^.

Our analysis found that several key metrics identified distinct driving patterns between older adults in the CN group and those in the ICI group. Table 3 summarizes the mean, median, and variance of these metrics, alongside the p-values from the Mann–Whitney U-test to evaluate the statistical significance of the observed differences. To visually summarize the observed differences in driving behaviours, refer to Fig. 1, which contrasts the distributions of key metrics between the Cognitively Normal and Incident Cognitive Impairment groups.

**Table 3:** Summary of statistical analysis for mobility metrics.

	Metric	Cognitively normal group (CN)		Incident cognitive impairment group (ICI)		p-value
		Mean	Standard deviation	Mean	Standard deviation	MW test
1	Cycle trips	0.1663	0.1752	0.1298	0.0776	0.2062
2	New destinations	0.2043	0.1163	0.209208962	0.1564	0.4285
3	Most common destination	0.0800	0.0456	0.083246799	0.0414	0.5591
4	Most common route	0.8240	0.1927	0.9123	0.0903	0.0942
5	Portion of trips to new destinations recognized as risky trips	0.1715	0.1188	0.2120	0.1557	0.5450
6	Portion of trips to most common destination recognized as risky trips	0.0985	0.15977	0.0711	0.0784	0.9706
7	Portion of trips to most common route recognized as risky trips	0.0977	0.1609	0.0711	0.0795	0.9799
8	Number of distinct paths used for most common destination	1.2886	1.0909	0.6892	0.3297	0.0061

### Findings from data

Before normalization, we observed a difference in the variety of routes to participants’ most common destination, with the CN group displaying a greater variety of routes (mean CN = 6.496) compared to the ICI group (mean ICI = 3.714). However, the Mann–Whitney U-test yielded a p-value of 0.017, which does not meet the threshold of significance set at $\alpha$ = 0.0062. After normalization, this pattern became more pronounced. For the number of distinct paths used for most common destination, the CN group had a higher mean (1.288) compared to the ICI group (0.689), with a Mann–Whitney p-value = 0.0061 indicating a significant difference after normalization.

Furthermore, this diminished variation in routes suggests that individuals with cognitive impairments may rely more on familiar and routine paths, possibly as a compensatory strategy to cope with navigational challenges. This finding underscores the potential need for interventions that could support route navigation and enhance spatial orientation in this population.

Upon applying the Bonferroni-corrected threshold of $\alpha = 0.0062$, no significant differences were detected in the frequency of visits to the first common destination or the most traveled route to this location. This indicates that routine driving behaviours, such as the frequency of visits and preferred routes, remain consistent across both groups, regardless of cognitive status.

### Impact of gender and rush hour on driving behaviours

We conducted separate analyses based on gender to investigate potential differences in behaviour patterns between men and women. We found that while there was a significant difference in the variety of routes used for the most common destination among women (p-value = 0.003), indicating that women in the ICI group took considerably more varied routes when traveling to their most common destination, no such difference was found among men (p-value = 0.008). Additionally, for the remaining measures, no significant differences were observed in either men or women, which is consistent with the overall results.

Furthermore, we examined the influence of rush hour driving versus non-peak times to determine its impact on the risk profiles associated with driving behaviours. This facet of our analysis did not yield any significant differences in risky trips between groups during rush hours or other times, suggesting that the timing of travel, in terms of peak and off-peak hours, may not deferentially affect the driving risk profiles within our study population.

## Discussion

This study systematically analyzed the navigational driving behaviours of older adults with and without incident cognitive impairment. We focused on the repeatability of their chosen destinations and routes, as well as the frequency of trips exhibiting specific characteristics such as cycle trips. This analysis aimed to assess differences in risk-taking and self-regulation behaviours between the two groups. We evaluated measures such as the introduction of new destinations, which could indicate risk-taking, and the frequency of repetitive cycle trips, which could suggest aimless driving. These measures represent behavioural differences between older adults with and without incident cognitive impairment. Additionally, the frequency of trips to a preferred destination and the tendency to select the most travelled route for this destination could reflect more cautious and self-regulated driving behaviours. Our study of navigational decisions among older adults has provided insights into driving behaviour that goes beyond mere transportation. It suggests that changes in driving patterns may serve as early indicators of lifestyle contraction in individuals with incident cognitive impairment.

The ICI group’s diminished variety of routes to the most common destination suggests an aversion or inability to process the complexities of changes occurring in novel environments. However, the maintained frequency of visits to common destinations and routes reveals a preservation of routine, a finding consistent with previous research that suggests daily functioning is not always immediately impacted by early cognitive impairment^22^. The routine may persist until cognitive decline progresses into the prodromal stages of the respective dementia etiology.

We also investigated risky behaviours associated with each trip to a new destination, along the most common route, and to the most common destination. Our goal was to assess whether the familiarity of the route and destination to the driver might influence the prevalence of risky behaviours^23^, and affect the two populations differently. Additionally, we explored whether rush hour impacts these risk profiles. Our findings indicate that the proportion of risky trips associated with new destinations and common routes did not differ significantly between the ICI and CN groups, implying that drivers with ICI do not inherently pose a greater risk on the road^24^. Additionally, implementing a longitudinal study design would allow us to closely monitor how driving behaviours change as cognitive impairment progresses. This approach would enable us to identify the specific stages at which certain behaviours become more pronounced. With this knowledge, we can tailor interventions to enhance road safety and support the independence of drivers.

However, our study has limitations that may impact the interpretation of the findings. Conducted solely in St. Louis, the results are not universally applicable due to regional driving variations. The unequal group sizes could also affect the statistical power of the comparisons made. The granularity of our data was limited by the low-frequency 30-s data collection rate, which constrained our feature extraction and, by extension, the depth of our behavioural analysis. Our focus on only a few risky driving behaviours did not capture the full spectrum of what constitutes driving complexity.

Additionally, we did not collect data on whether participants used navigation assistance, a factor that could significantly affect driving behaviours. The role that medication and other health interventions might play in mitigating or exacerbating driving risks in older adults with cognitive impairments was not explored. This oversight could be crucial, as these interventions can have profound impacts on cognitive and motor functions, thereby affecting driving abilities. Moreover, Factors such as socioeconomic status, urban vs. rural driving contexts, and psychological conditions like anxiety, which can all influence driving behaviours, were also not considered^25^.

Despite these limitations, the study successfully characterizes navigational behaviours and decision-making patterns among older drivers, shedding light on the dynamics of destination choices, trip repeatability, and openness to new driving environments. These are crucial for understanding the driving habits associated with cognitive impairments and recognizing the unique needs of this population. Prior research has linked aging and cognitive decline to changes in driving behaviour. However, our study’s contribution lies in examining how these factors influence destination choices, trip repeatability, and adaptability to new driving environments.

In conclusion, while the study sheds light on certain patterns of driving behaviour in the context of ICI, it also highlights the need for further research. Future studies should aim for a broader demographic and geographic scope, a higher resolution of data collection, and a more extensive array of influencing factors. Enhancing our understanding in these areas is essential for crafting supportive interventions that promote safety and independence for older adults with cognitive impairments and for developing driving as a behavioural biomarker for dementia.

## Data Availability

The datasets used and/or analyzed during the current study are available from the corresponding author upon reasonable request.
